# Consistency of Seasonal Mean and Extreme Precipitation Projections Over Europe Across a Range of Climate Model Ensembles

**DOI:** 10.1029/2022JD037845

**Published:** 2023-01-04

**Authors:** N. Ritzhaupt, D. Maraun

**Affiliations:** ^1^ Wegener Center for Climate and Global Change University of Graz Graz Austria

## Abstract

Uncertainties of regional precipitation projections are substantial, and users of such projections face the so‐called practitioners dilemma: a plethora of projections with different models from different ensembles of different types and generations are available. But the consistency of these projections has not been systematically assessed, such that no clear guidance about the use of these ensembles exists. Therefore, we systematically compare, separately for each season, projections of mean precipitation and extremes of daily precipitation over Europe across a wide range of climate model ensembles. We specifically consider the global climate model ensembles CMIP3, CMIP5, Coupled Model Intercomparison Project Phase 6 (CMIP6), and HighresMIP as well as the regional climate model ensembles ENSEMBLES and EURO‐CORDEX. All considered ensembles agree in their large‐scale patterns of changes for both mean and extreme daily precipitation, but at the regional scale, substantial discrepancies and inconsistencies are evident. Within and across ensemble spread is strongest in summer, in particular for the drying trend over the Mediterranean and Eastern Europe. CMIP5 and CMIP6 are broadly consistent. The regional climate model (RCM) ensembles modify the signals of the driving global climate models indicating potential added value. The high resolution of the RCM and HighresMIP ensembles seems to be key over the Alps for summer precipitation. Our study provides important information for users of climate projections as it helps to establish continuity across generations and types of climate models, and aids the design of new climate impact studies.

## Key Points

- Identification of robust and conflicting projections of mean and extreme precipitation across Europe in six climate model ensembles
- For mean precipitation changes, ensembles differ in the strength of Mediterranean and south‐eastern European summer drying
- For extreme precipitation changes, regional climate models often alter the changes projected by their driving global models

## 1 Introduction

The response of the Earth's hydrological cycle to global warming is one of the greatest concerns regarding the effects of climate change (Giorgi et al., 2019). Globally, the water cycle and hence global‐mean precipitation is well understood and primarily constrained by the energy budget where radiative cooling of the troposphere and heating of the surface is balanced by upward heat fluxes (causing precipitation) (Allen & Ingram, 2002; Manabe & Wetherald, 1975). But regional changes in precipitation are much more uncertain (Shepherd, 2014). While the increase in atmospheric water vapor is well constrained by the Clausius‐Clapeyron relation and amounts to roughly 7% K^−1^ at constant relative humidity (Trenberth et al., 2003), the actual precipitation changes depend on much more uncertain changes in the atmospheric circulation (Pfahl et al., 2017; Shepherd, 2014).

Information about possible future changes in regional precipitation mainly stems from projections using climate model ensembles (Doblas‐Reyes et al., 2021). Roughly in synchrony with the major assessment reports of the Intergovernmental Panel on Climate Change (IPCC), new ensembles of coupled global general circulation models (GCMs) and Earth system models (ESMs) have been generated, with the Coupled Model Intercomparison Project Phase 6 (CMIP6, Eyring et al., 2016) being the most recent generation available. Over Europe, both the CMIP3 (Meehl et al., 2007) and CMIP5 (Taylor et al., 2012) ensembles have been dynamically downscaled to better represent regional processes, resulting in the ENSEMBLES (van der Linden & Mitchell, 2009) and EURO‐CORDEX (Jacob et al., 2014, 2020) regional climate model (RCM) ensembles. Additionally, more experimental model ensembles exist such as the CMIP6‐endorsed HighresMIP ensemble of high resolution GCMs (Haarsma et al., 2016).

All these ensembles have been, or can in principle, be used for climate risk assessments and regional adaptation planning (Doblas‐Reyes et al., 2021; Ranasinghe et al., 2021). But while all these ensembles agree in their main continental‐scale change patterns of precipitation, even a superficial comparison between them reveals substantial differences at the regional scale (Coppola et al., 2021; Jacob et al., 2014; Maraun, 2013). These discrepancies pose serious questions for users of regional climate model output (Barsugli et al., 2013): Which model ensemble(s) should be used for a given use‐case? Are previous studies based on older climate models still valid, or do they have to be considered outdated? Often, the a priori assumption is that newer generations better represent relevant meteorological processes and thus provide more reliable projections (e.g., Jacob et al., 2014; Scoccimarro & Gualdi, 2020), but the actual added value of new ensembles for projecting regional climate is not always evident, in particular without in‐depth process‐based evaluations (Doblas‐Reyes et al., 2021). This problem has been coined the practitioner's dilemma (Barsugli et al., 2013): users are left with a plethora of potentially contradictory model projections, without clear guidance about which of these models to use.

Here, we do a first step in providing such guidance and conduct a systematic comparison of a wide range of climate model ensembles with respect to precipitation projections over Europe. Given its relevance for climate risk assessments (Ranasinghe et al., 2021), we do not only consider changes in seasonal mean precipitation, but also in extreme precipitation as measured by 20‐season return levels of daily precipitation. We consider the global CMIP3, CMIP5, CMIP6, and HighresMIP ensembles, as well as the regional ENSEMBLES and EURO‐CORDEX ensembles. Our study thus greatly expands the results by Fernández et al. (2019) who studied changes in mean temperature and precipitation over Spain.

The overall aim of our study is to identify regions of robust projections, where different ensembles simulate consistent changes, and regions where different ensembles provide substantially different projections. Differences between projections from different climate model ensembles result from inter‐ensemble model differences (i.e., differences that are common to all models within an ensemble), internal variability and the scenarios used for each ensemble. Our focus is on climate model differences. Hence, we minimize the other sources of uncertainty by (a) defining changes between the end of the 21st century and 20th century for high emission scenarios to reduce the influence of internal variability; and (b) by expressing changes per Kelvin warming to approximately remove the influence of different scenarios. A comprehensive analysis of the causes of climate model differences is far beyond the scope of this study.

## 2 Data and Methods

### 2.1 Data

We analyze simulations from the following climate model ensembles: The GCM ensembles CMIP3 (Meehl et al., 2007), CMIP5 (Taylor et al., 2012), CMIP6 (Eyring et al., 2016) and the CMIP6‐endorsed HighresMIP (Haarsma et al., 2016), as well as the RCM ensembles ENSEMBLES (van der Linden & Mitchell, 2009) and EURO‐CORDEX (Jacob et al., 2014, 2020), with boundary conditions from CMIP3 and CMIP5 respectively. To aid the comparison of GCM and RCM ensembles, we additionally consider subsets of those CMIP3 and CMIP5 GCMs which have been used as boundary conditions for ENSEMBLES and EURO‐CORDEX.

The resolutions of the considered models from CMIP3 (16 models considered), CMIP5 (24 models), and CMIP6 (27 models) increasing from about 300 to 150 km to 100 km, respectively. In HighresMIP, models of different resolution have been compared. We restrict our analysis to the four available high‐resolution models with a horizontal resolution of approximately 50 km. From the ENSEMBLES project, 14 models with a 25 km resolution have been considered, and 39 models from EURO‐CORDEX with a resolution of 12 km.

The GCM generations have been forced with different emission scenarios. To minimize the influence of internal variability, we have used the strongest scenario for most ensembles: the SRES A1B scenario for CMIP3 and ENSEMBLES, the RCP8.5 scenario for CMIP5 and EURO‐CORDEX, and the SSP5‐8.5 scenario for CMIP6. In the case of the SRES scenario, we did not use the strongest scenario A2 but instead A1B because of data availability and we aim to include as many models of each ensemble as possible. To enable a direct comparison of the different scenarios, we have expressed the simulated changes per degree Kelvin warming (see below).

### 2.2 Methods

Our study aims at comparing climate model uncertainties across different ensembles in precipitation projections over Europe. We considered seasonal means and extremes of daily precipitation for boreal winter (DJF), spring (MAM), summer (JJA), and autumn (SON). Extreme precipitation is defined as 20‐year return values of each considered season separately (see Appendix A for the estimation of these values using a block maxima approach).

To increase the signal‐to‐noise ratio (i.e., to reduce the effect of internal variability), we calculate change signals from two far apart baseline (CTRL) and future (SCEN) periods, different for each specific ensemble: 2081–2100 versus 1981–2000 for CMIP3; 2071–2100 versus 1971–2000 for CMIP5, CMIP6, ENSEMBLES and EURO‐CORDEX; and 2021–2050 versus 1950–1979 for HighresMIP. For CMIP3, only 20‐year time slices were available. Because of internal variability, this shorter period slightly deteriorates the signal‐to‐noise ratio compared to the other ensembles (see discussions below). To enable a direct comparison of the different scenarios used (and, as a positive side effect, to approximately eliminate the effect of the different periods), we have expressed each signal as a change per Kelvin warming. This approach has been widely used in the most recent IPCC report (Chen et al., 2021) and is usually expressed in terms of global mean surface temperature change. As the RCMs do not provide global mean temperature changes, we have instead calculated changes relative to European mean warming as follows. Separately for each season, we calculate climate change signals (CCS) by normalizing the change in mean or extreme precipitation between the CTRL and SCEN periods, Δ*P* = *P* ^*SCEN*^−*P* ^*CTRL*^, by the corresponding precipitation statistic of the CTRL period. This ratio is divided by the annual European mean temperature change (Δ*T*) between the same reference periods to obtain the CCS in percent per degree warming:

(1) $$CCS=\frac{\Delta P}{P^{\mathrm{CTRL}}}\cdot \frac{1}{\Delta T}\cdot 100\%$$

This normalization with mean warming furthermore approximately removes differences in the representation of climate sensitivity by the GCMs, which is a major source of uncertainty in climate projections (Chen et al., 2021). All calculations are carried out on the native grids, and where necessary, the results are interpolated to the EURO‐CORDEX grid for the comparison of the ensembles.

An initial analysis considers multi model mean changes for each of the different ensembles. Robustness and significance of changes within each ensemble is assessed similar to the method described in the recent IPCC assessment report (Gutiérrez et al., 2021): CCS are considered robust if at least 80% (75% for HighresMIP) of the models within one ensemble agree on the sign of change. Further, the ensemble is considered simulating significant changes if the CCS of at least 66% of the models are significant. Here, significance for a single model is assessed using a *t*‐test with a 90% significance level, that is, the simulated change exceeds $\gamma =\sqrt{\frac{1}{N}}\cdot 1.645\cdot \sigma$, where *σ* is the seasonal interannual standard deviation of the historical period and *N* denotes the length of the time slices in years. We apply the same significance test also for changes in extreme precipitation, by considering changes in seasonal maxima. This significance test is only approximately conservative as block maxima are not normally distributed. Moreover, this test measures significance in changes in the expected seasonal maxima and not directly in return values. As our main aim is to identify (in‐)consistencies between ensembles, we have chosen the hatching—different to the IPCC—to highlight robustness within ensembles.

To quantitatively compare the different ensembles, we conduct two analyses of the robustness of the projected changes. First, we consider the distribution of area mean CCS across eight European sub‐regions. These regions have been defined in the PRUDENCE project (Christensen & Christensen, 2007). For each region, we summarize the CCS distribution separately for each ensemble by box‐whisker plots, where the boxes cover the 25th and 75th percentiles with the ensemble median in between and the whiskers indicate the minimum and maximum CCS of the corresponding ensemble.

Second, we investigate the robustness of the change signals between the ensembles at the grid‐scale using pairwise Gaussian overlaps. The overlap of two probability density functions *f*(*x*) and *g*(*x*) is defined as *∫* min(*f*(*x*), *g*(*x*))*dx*. We estimate overlaps between different ensembles assuming that the CCS of the models within one ensemble are approximately normally distributed, $N\left(\mu ,\sigma^{2}\right)$, with expectation *μ* and standard deviation *σ*. This measure was compared with the Anderson‐Darling test, which provided similar results (see Supporting Information S1).

## 3 Results

### 3.1 Mean Precipitation

**Figure 1:**
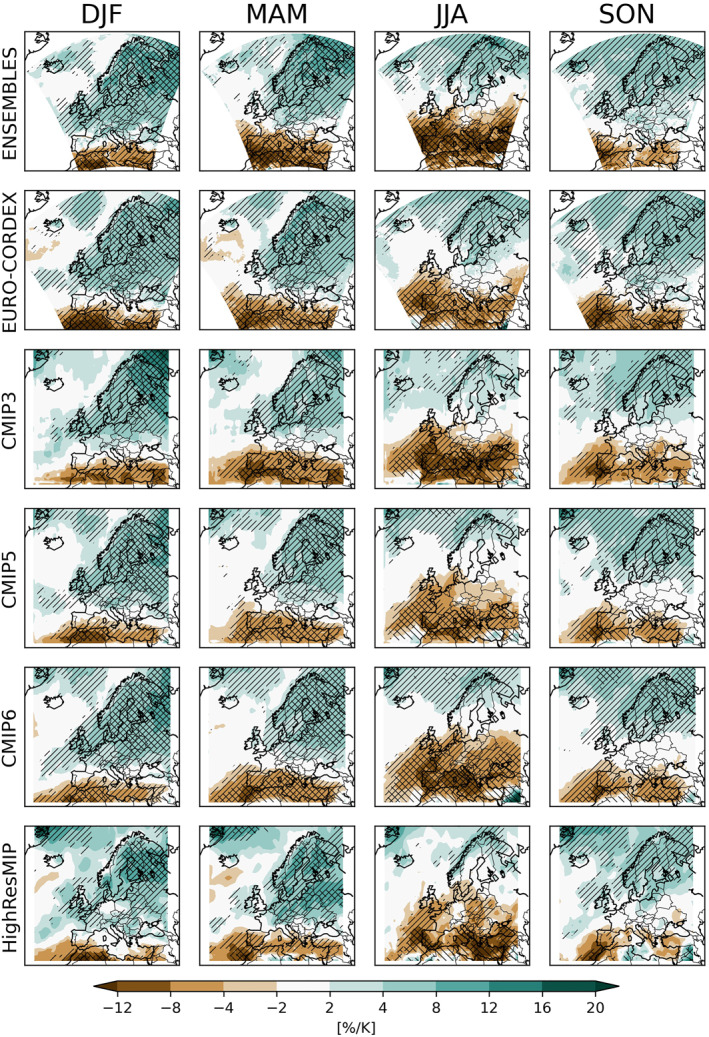
Ensemble medians of seasonal mean precipitation changes per degree warming [% K^−1^]. Hatching indicates areas with robust (“//”) and significant (“∖∖”) change signals (see text for details).

The main patterns of seasonal mean precipitation changes are well known (Christensen & Christensen, 2007; Coppola et al., 2021; Gutiérrez et al., 2021; Jacob et al., 2014; Maraun, 2013; Rajczak & Schär, 2017; Rajczak et al., 2013; van der Linden & Mitchell, 2009), can be explained by basic physical processes (Brogli et al., 2019; Douville et al., 2021; Seager et al., 2014; Trenberth et al., 2003; Zappa et al., 2013, 2015) and broadly agree across all ensembles (Figure 1; ensemble medians are shown to eliminate the effect of individual outliers): precipitation is expected to increase over northern Europe, and to decrease over southern Europe, with a seasonally shifting transition region. At closer inspection, however, differences between the ensembles become evident. The most apparent discrepancy is the varying magnitude of summer drying over the Mediterranean: CMIP5 and EURO‐CORDEX show a weaker drying than all other ensembles. Additionally, the regional position of the transition zone varies substantially between ensembles. For instance, ENSEMBLES projects no median change in spring precipitation over northern France, Belgium and Great Britain, while EURO‐CORDEX projects a weak but robust wetting. Similarly, ENSEMBLES projects a weak but robust summer drying from parts of western Germany to south Eastern Europe, where EURO‐CORDEX projects no change. Median changes of CMIP3, CMIP5, and CMIP6 are largely similar, apart from the magnitude of Mediterranean summer drying. HighresMIP broadly agrees with the CMIP ensembles. Regional differences might be caused by the rather small sample of only four models. The change signals are robust over most regions where the change is at least ±4% K^−1^. Significant changes are restricted to Scandinavia in DJF and MAM, and southern Europe in MAM and JJA for most ensembles. Comparing all ensembles, two key differences can be found: first, the strength of the Mediterranean summer drying depends strongly on the chosen ensemble. And second, over south Eastern Europe most ensembles project a median drying, whereas EURO‐CORDEX projects no change (and the driving CMIP5 ensemble projects a non‐robust non‐significant drying only). The effect of the shorter time period for CMIP3 is hardly visible, resulting in slightly smaller and more patchy robust and significant areas compared to CMIP5 and CMIP6. An important question is whether RCMs modify the change signals of their driving GCMs. As Figure 1 shows the full CMIP3 and CMIP5 ensembles, but only a subset of them has been downscaled, a comparison would be misleading. A comparison of RCM ensembles only with their driving ensembles can be found below.

**Figure 2:**
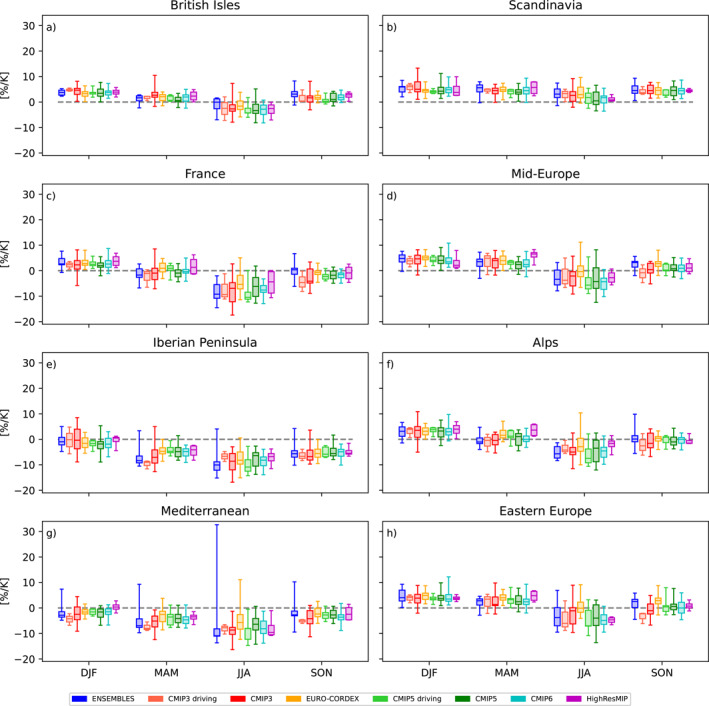
Distribution of area mean changes in seasonal mean precipitation, separately for each ensemble [% K^−1^]. Each panel shows the results for one European sub‐region. The distribution of each ensemble is summarized by a box‐whisker plot. The ensemble median is indicated as bold line, the boxes cover the 25th to 75th percentile range, the whiskers depict the minimum and maximum values respectively. For CMIP3 and CMIP5 the full range of models is shown as well as the corresponding sub‐ensembles used to drive the ENSEMBLES and EURO‐CORDEX RCMs, respectively.

To characterize the consistency of ensembles beyond the comparison of medians, we compare the full ensemble spread. This is done for the area mean changes in seasonal mean precipitation, separately for all PRUDENCE regions (Figure 2). Overall, the ensembles by‐and‐large agree. In particular, at the scale of the considered (large) regions, most ensembles agree in sign. The highest robustness is trivially found far away from the transition regions, for example, for Scandinavia in all seasons but summer, for the British Isles in winter, and for Spain in all seasons but winter. The strongest spread within each ensemble and across ensembles appears in summer. To assess systematic modifications of the CCS from the driving GCMs by the corresponding RCMs, we show boxplots both for the full CMIP3 and CMIP5 ensembles as well as for the sub‐ensembles of GCMs which have been downscaled in ENSEMBLES and EURO‐CORDEX respectively. For both ensembles, the RCMs modify the GCM changes in a similar way. This modification is most prominent in autumn, where the RCMs alter GCM changes toward more positive values in many regions. The deviation of EURO‐CORDEX summer precipitation changes from all other ensembles in south Eastern Europe is evident also in the change distribution for Eastern Europe.

**Figure 3:**
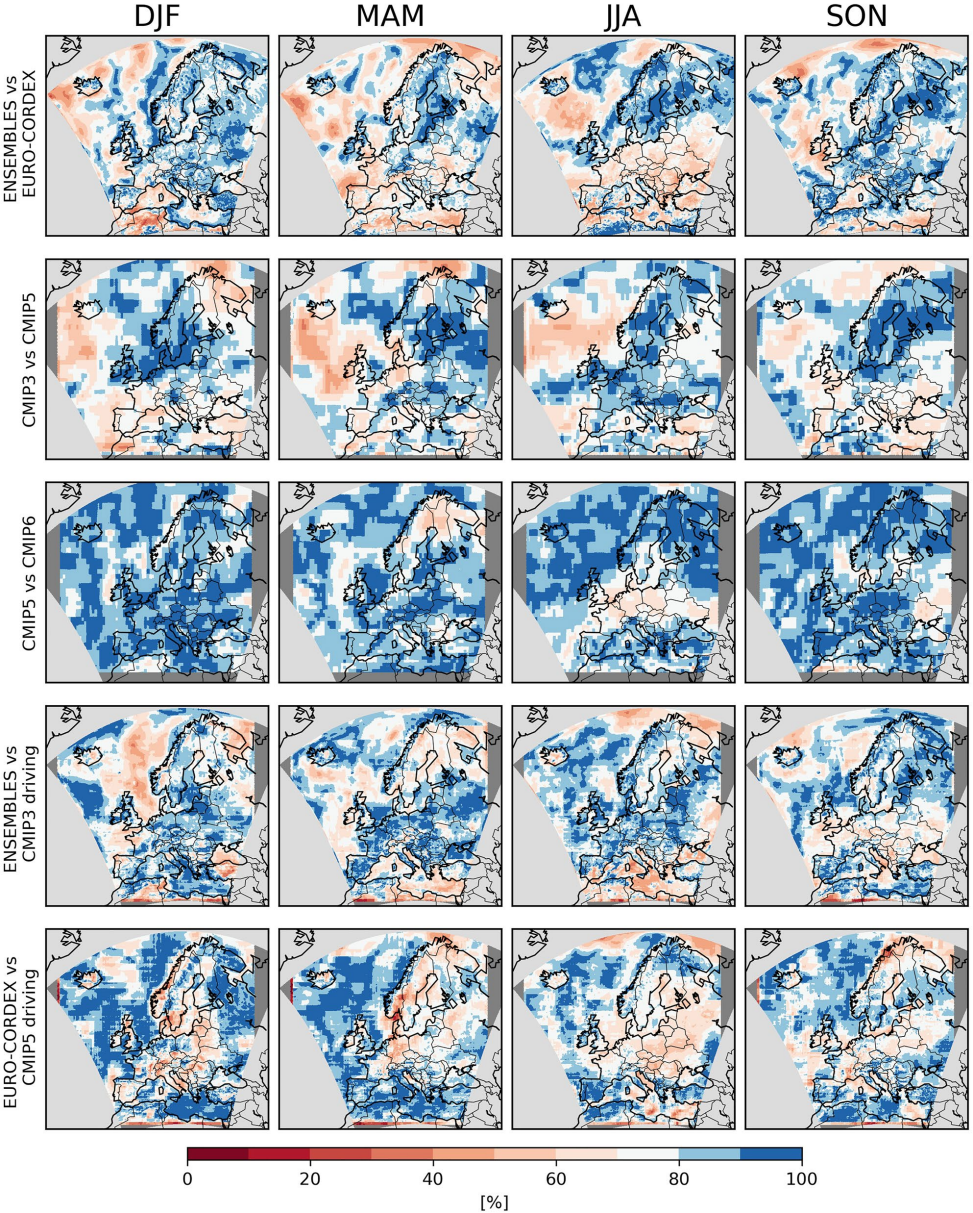
Consistency of projected seasonal mean precipitation between the considered ensembles. Each panel shows Gauss overlaps [%] of the ensemble distributions of changes in seasonal mean precipitation. Each row depict one pairwise comparison between two ensembles.

In general, however, the PRUDENCE regions are too large to capture the regional differences visible in Figure 1. We therefore employ the Gauss overlap as a robustness measure that collapses the consistency of two ensembles to one number and thus can be presented as a spatial map (Figure 3). The results broadly agree with those for the ensemble medians (Figure 1), suggesting that much of the inconsistency between ensembles can be explained by relative shifts of the ensembles. For ENSEMBLES and EURO‐CORDEX, the largest inconsistencies occur in spring (mainly Iberian Peninsula, France, southern UK and Eastern Europe) and summer (mainly Mid‐ and Eastern Europe and Turkey). Inconsistencies between CMIP3 and CMIP5 are evident for all seasons; for CMIP5 versus CMIP6 they are strongest in spring over Scandinavia and summer over Mid‐Europe. The ENSEMBLES RCMs show the most severe inconsistencies with their driving CMIP3 GCMs during spring over Scandinavia, and autumn over central and Eastern Europe. For EURO‐CORDEX, the inconsistencies with respect to their driving CMIP5 GCMs are much stronger, in particular in central and Eastern Europe for winter, spring and summer. HighresMIP has not been considered in this analysis because of the low number of available models.

### 3.2 Extreme Precipitation

**Figure 4:**
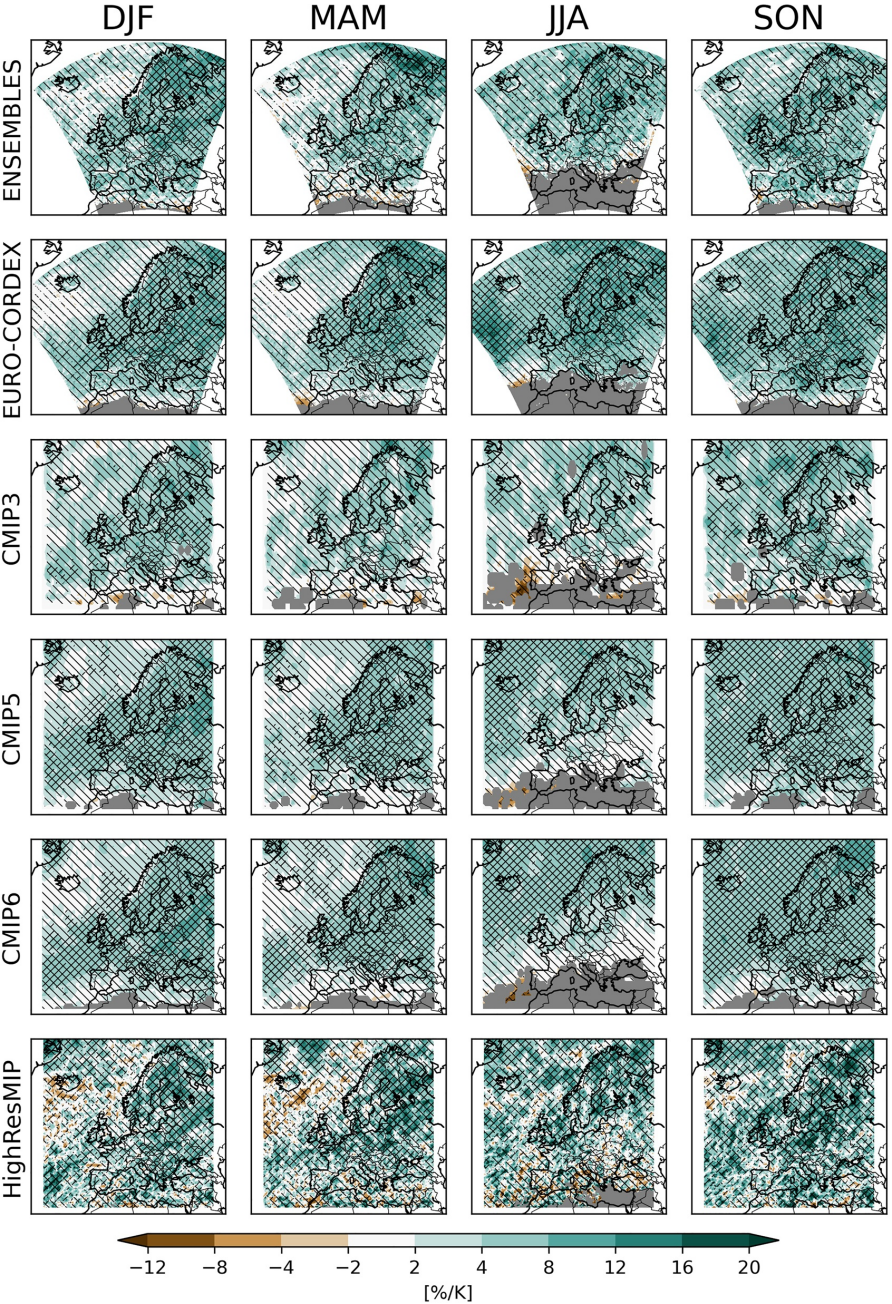
Same as Figure 1, but for seasonal extremes of daily precipitation. Grid‐points, where the estimated return values where unrealistically high, are marked in gray.

For daily precipitation extremes, expressed as 20‐season return values (see Appendix A), we again reproduce the large‐scale patterns known from the literature (Coppola et al., 2021; Jacob et al., 2014, 2020; Rajczak & Schär, 2017; Rajczak et al., 2013; Scoccimarro et al., 2016; van der Linden & Mitchell, 2009). Projected median changes are positive or negligible almost everywhere (Figure 4), highlighting the dominant role of the Clausius‐Clapeyron relationship for extreme precipitation over many European regions. Although the sign of the signal is broadly consistent and robust, the actual magnitude differs between ensembles, with EURO‐CORDEX simulating the strongest increases. Depending on the ensemble, the changes are significant over many regions. The shorter time period for CMIP3 results in smaller and more patchy significant areas compared to CMIP5 and CMIP6.

**Figure 5:**
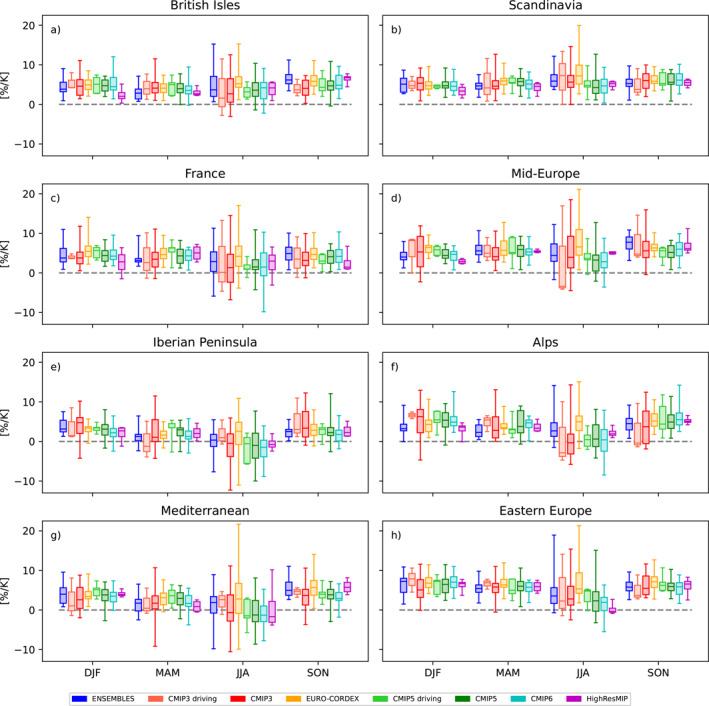
Same as Figure 2 but for seasonal extremes of daily precipitation.

Mean changes in daily precipitation across the PRUDENCE regions (Figure 5) show a much stronger within‐ and across‐ensemble spread than for changes in mean precipitation, in particular for the summer season. Nevertheless, the sign of changes is broadly consistent across most ensembles for most regions and seasons. Exceptions are the Mediterranean and the Iberian Peninsula, but recall that here large parts of the regions have not been considered because of the bad fit of the GEV distribution. Over the Alps, a clear dependence on model resolution is evident: while all standard resolution GCMs project zero changes in daily summer precipitation extremes, the RCM ensembles ENSEMBLES and EURO‐CORDEX and the high resolution GCMs from HighResMIP all simulate increases in these events. This result corroborates the findings of Giorgi and Gutowski (2016), who found that RCMs more realistically simulate changes in summertime convective precipitation over the Alps due to enhanced potential instability by high‐elevation surface heating and moistening.

**Figure 6:**
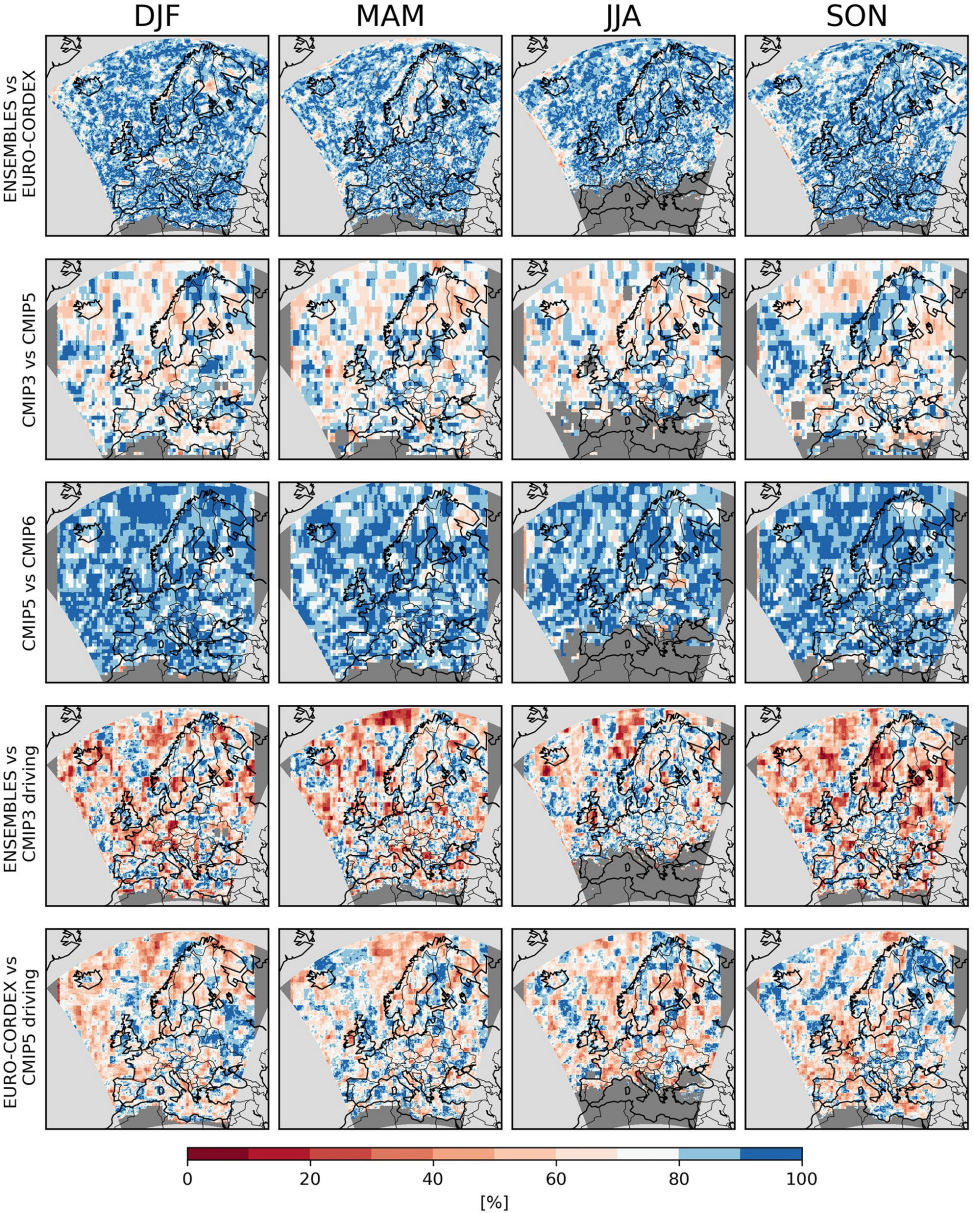
Same as Figure 3 but for seasonal extremes of daily precipitation.

Local pairwise consistency between ensembles (Figure 6, again measured by Gaussian overlaps) show remarkable differences between the different pairwise comparisons. The ENSEMBLES and EURO‐CORDEX ensembles are broadly consistent, even though the GCM ensembles (albeit considering all models, not just the driving ones) are not: the patterns of consistency between CMIP3 and CMIP5 are rather patchy. In general this patchiness may arise from model differences as well as from sampling uncertainties related to the low number of extreme events considered in the pointwise comparisons. No effect of the shorter CMIP3 time slice is evident. But the fact that these inconsistencies are much stronger than between CMIP5 and CMIP6 indicates that CMIP3 indeed deviates from the other CMIP ensembles because of model differences rather than sampling uncertainty. Both ENSEMBLES and EURO‐CORDEX are strongly inconsistent with their driving GCM ensembles, in particular over Scandinavia and north Eastern Europe in autumn. Considering the broad agreement of the two RCM ensembles, these inconsistencies hint at a potential added value of the RCMs at simulating changes in daily extreme precipitation.

## 4 Discussion and Conclusion

We have assessed the robustness of projected changes in mean and daily extreme precipitation across four GCM and two RCM ensembles. Projected changes have been expressed per Kelvin European mean warming to approximately remove uncertainties in the representation of climate sensitivity and to enable a comparison across different generations of forcing scenarios.

All considered ensembles agree in their large‐scale patterns of changes in mean and extreme daily precipitation, but at the regional scale, substantial discrepancies and inconsistencies are evident. For seasonal mean precipitation, most ensembles agree in sign for most PRUDENCE regions. Within‐ and across‐ensemble spread is strongest in summer, and the most prominent inconsistencies are: (a) the strength of Mediterranean summer drying, where CMIP5 and EURO‐CORDEX project a weaker change than all other ensembles; and (b) the summer drying over Eastern Europe, where EURO‐CORDEX projects no change, but all other ensembles a drying. The RCMs tend to alter GCM changes toward wetter changes. Daily extreme precipitation is projected to increase over large parts of Europe (evidence for the Mediterranean is weak because of the low amount of non‐zero events), with varying magnitude across models and ensembles. Within‐ and across‐ensemble spread is higher than for mean precipitation, and especially high for summer. The strongest increases are found in the EURO‐CORDEX ensemble. CMIP5 and CMIP6 are broadly consistent, with notable differences to CMIP3. The ENSEMBLES and EURO‐CORDEX ensembles both modify the signals of the driving CMIP3 and CMIP5 models such that they are broadly consistent with each other. These modifications thus hint at potential added value of the RCMs at projecting regional changes in extreme precipitation. In particular over the Alps resolution may be important: all high resolution ensembles, ENSEMBLES, EURO‐CORDEX, and HighresMIP, project an increase in extreme summertime precipitation, whereas standard resolution GCMs project no changes. These findings are consistent with the results from Giorgi and Gutowski (2016), who found a better representation of summer convective rainfall over the Alps in RCMs rather than GCMs.

Our results provide important information for users of climate information. First, they help to establish continuity across generations and types of climate models, and thus also climate impact studies based on these models: where can impact studies relying on precipitation projections be considered “still valid” because the underlying climate model ensembles sample the known model uncertainty, and where are amendments required? Similarly, our results help to plan new impact studies: where does, for example, the EURO‐CORDEX ensemble comprehensively sample known climate model projections, and where do other ensembles have to be considered additionally?

Our study explores differences and does not provide any explanations of inconsistencies between ensembles. It does, however, point to cases—regions, seasons and aspects of precipitation—where further process based analyses are required to understand and reconcile these inconsistencies: can they be attributed to the representation of specific physical mechanisms? And if so, can some of these representations be ruled out as implausible? Such fitness‐for‐purpose studies could ultimately help to exclude in‐appropriate models and to understand where standard GCM projections may suffice, where the added value of RCMs or high resolution GCMs is necessary to generate credible projections, or where RCMs may even distort GCM signals. But such a comprehensive process‐based analysis is far beyond the scope of this study. As such, our study provides an initial step to resolve the practitioners dilemma, and thus contributes to the distillation of regional climate information. But as a next step, region specific and process‐based studies are required to understand the mechanisms underlying regional climate model biases (e.g., Maraun et al., 2021) and to assess the plausibility of regional climate projections (e.g., Giorgi & Gutowski, 2016). In particular, future studies should exploit newly available ensembles of convection permitting climate models (Ban et al., 2021; Coppola et al., 2020; Pichelli et al., 2021), which might substantially improve the representation of summer convective rainfall. Similarly, single model initial conditions ensembles, both using GCMs (Deser et al., 2020; Maher et al., 2019; Marotzke, 2019) and RCMs (Böhnisch et al., 2020; von Trentini et al., 2020), may substantially reduce sampling uncertainties for extreme events and should thus regularly be considered to characterize changes in such events.

## Conflict of Interest

The authors declare no conflicts of interest relevant to this study.

## Supporting information

Supporting Information S1

## Data Availability

All data that support the findings of this study are openly available. CMIP3 data can be accessed through https://esgf-node.llnl.gov/projects/cmip3/. CMIP5, CMIP6, HighresMIP, and EURO‐CORDEX data can be found at https://esgf-data.dkrz.de/projects/esgf-dkrz/. ENSEMBLES data are available at http://ensemblesrt3.dmi.dk/data/A1B/.

## Appendix A Return Value Estimation

A detailed introduction of statistics in extremes and the generalized extreme value (GEV) theory can be found, for example, in Katz et al. (2002).

The GEV theory is coupled with the block‐maximum approach, where blocks are defined and in the further analysis just the maximum value of each block is considered. In our analysis, the blocks consists of the 30 annual seasonal daily maximum precipitation values within the 30‐year (20‐year in case of CMIP3) periods (29 for DJF). The cumulative distribution function of the GEV distribution can be calculated as:

(A1)
$$\begin{array}{ccc} G\left(y\right) & = & \exp\left(-{\left[1+\xi \left(\frac{y-\mu }{\sigma }\right)\right]}^{-1/\xi }\right)\;for\;\xi \neq 0\\ G\left(y\right) & = & \exp\left(-\exp\left(-\frac{y-\mu }{\sigma }\right)\right)\;for\;\xi =0 \end{array}$$

It contains the location (*μ*), scale (*σ*), and shape (*ξ*) parameters and can take three types, which depend on the shape parameter. The types are the Gumbel (*ξ* = 0), Frechet (*ξ* > 0) and Weibull (*ξ* < 0) distributions. The three parameters are estimated using a maximum‐likelihood estimator.

After the estimation of the GEV parameters, one is able to calculate return values and associated return periods. The return values (*z*_*m*_) can be calculated using the inverse distribution function of the GEV

(A2)
$$\begin{array}{ccc} z_{m} & = & G^{-1}\left(1-\frac{1}{m}\right)\\ & = & \mu -\sigma \log\left(-\log\left(1-\frac{1}{m}\right)\right) \end{array}$$

where *m* corresponds to the desired return period. In summer, the calculation of return values provides unrealistically high values at some grid‐points in southern Europe resulting from a few very high values while the majority of values is zero. These many zero values lead to a too large shape‐parameter. Therefore, the grid‐points, where the shape‐parameter is outside the range of −1.5 and 1.5, are masked for the calculation of regional means.
