# Supplementary material for: Consistency of Seasonal Mean and Extreme Precipitation Projections Over Europe Across a Range of Climate Model Ensembles
Source: J Geophys Res Atmos. 2023 Jan 4;128(1):e2022JD037845. doi: 10.1029/2022JD037845 (PMC10078419; doi:10.1029/2022JD037845)
Supplement: Supplementary file 1 — Supporting Information S1 [file JGRD-128-0-s001.pdf]

AGU PUBLICATIONS

JGR: Atmospheres

Supporting Information for

Consistency of seasonal mean and extreme precipitation projections over Europe across a range of climate model ensembles

N.Ritzhaupt [1], D.Maraun [1]

[1]Wegener Center for Climate and Global Change, University of Graz, Graz, Austria

Contents of this file

Figures S1 to S3
Tables S1 to S6

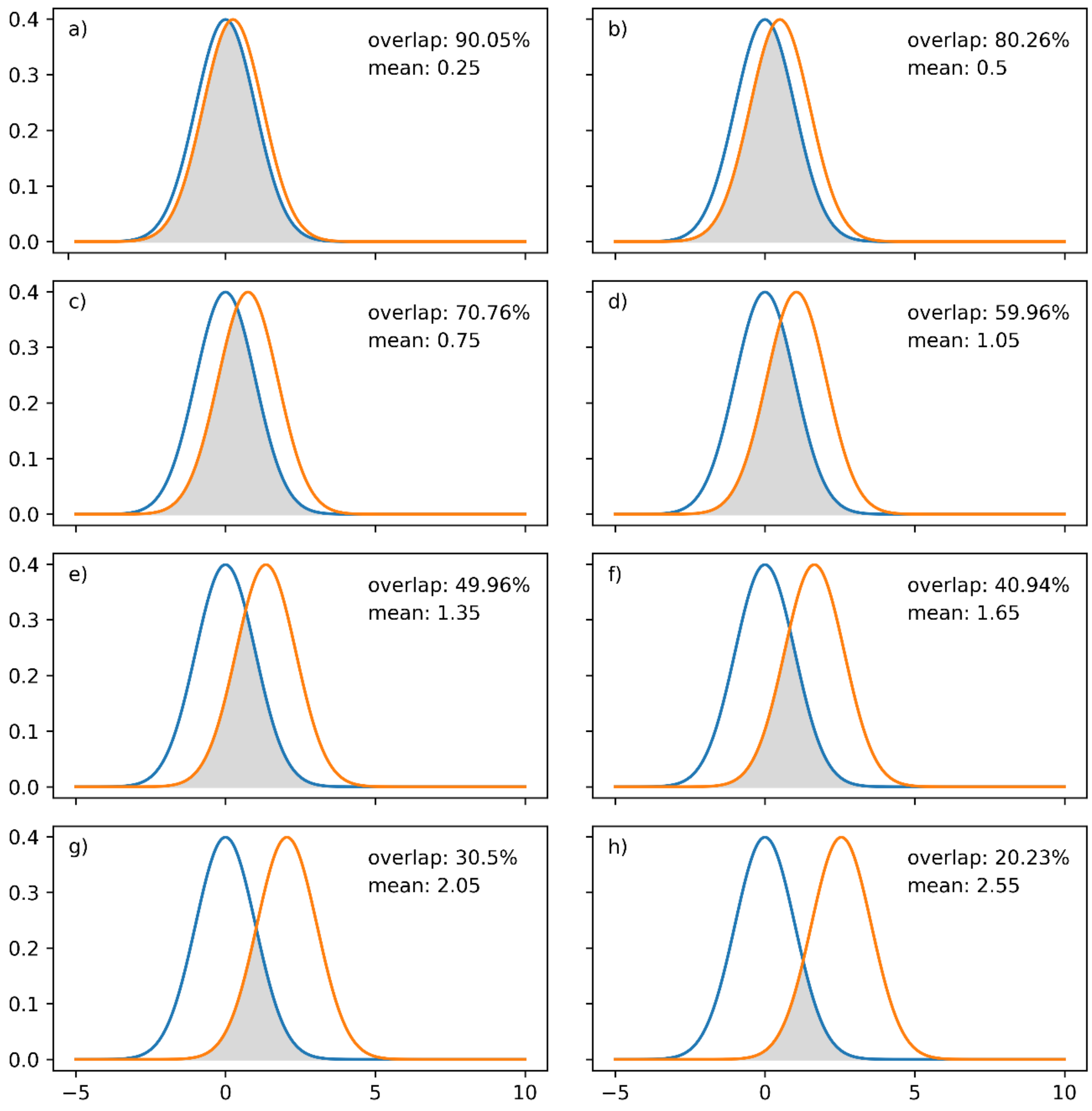

Figure S1. Concept of Gaussian overlaps. The first probability density function (pdf) is calculated with mean=0 and standard deviation (std) of 1 (blue curve). The second pdf (orange curve) has the same std. The overlaps in 10-percent steps are shown and the corresponding mean values of the second pdf are given. The overlap area of both pdfs is highlighted in grey. The overlap of 80% (threshold of high agreement) corresponds to half a standard deviation.

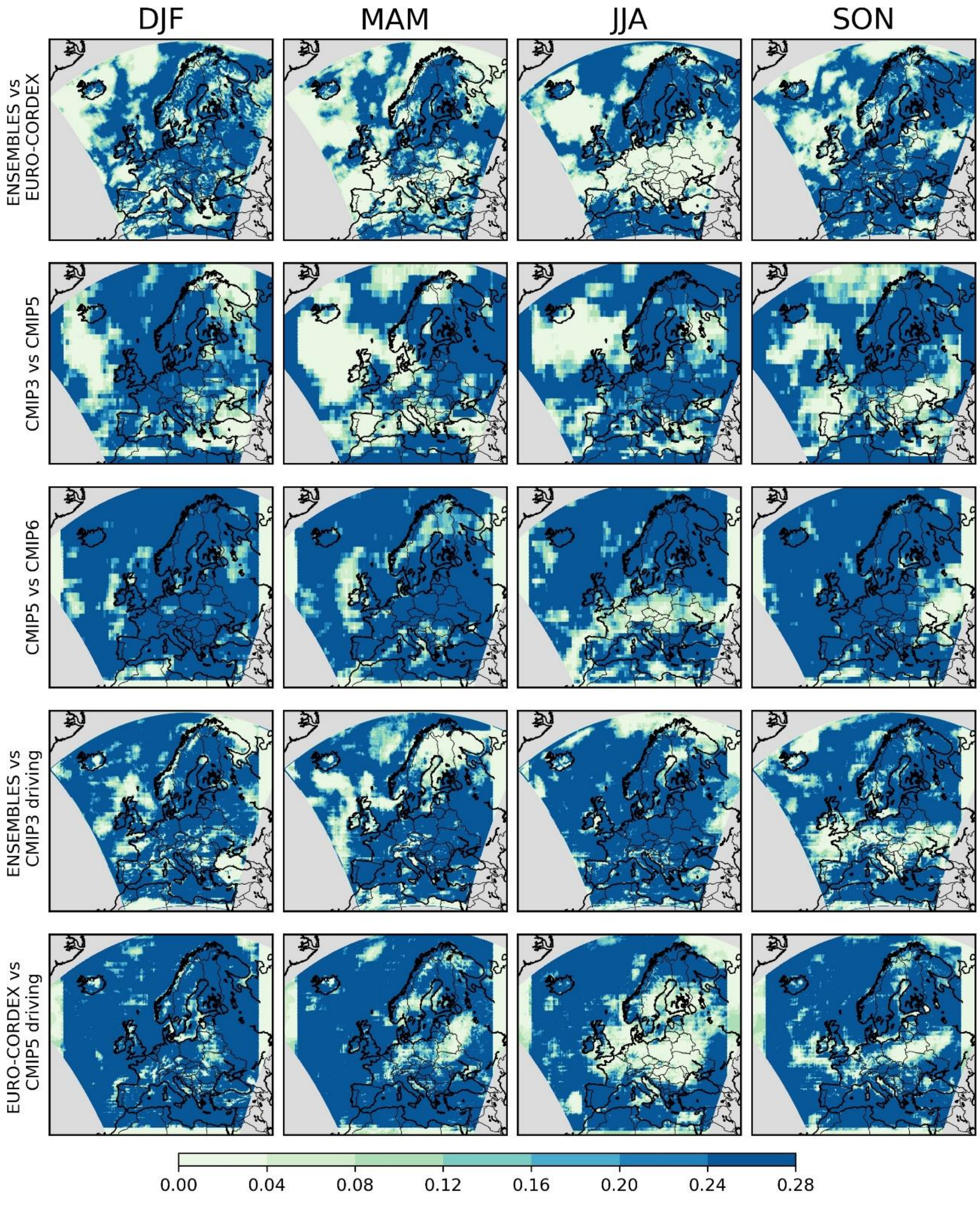

Figure S2. The Anderson-Darling test of pairwise ensemble combinations as a supplementary measure of robustness for mean precipitation. The colorbar shows the p-value of the test statistic and significant differences between the ensembles appear with a low p-value (light colors). Same combinations as in the Gaussian overlap plots are

shown. The main areas with significant differences are the same for both robustness measures (Gaussian overlaps and Anderson-Darling test).

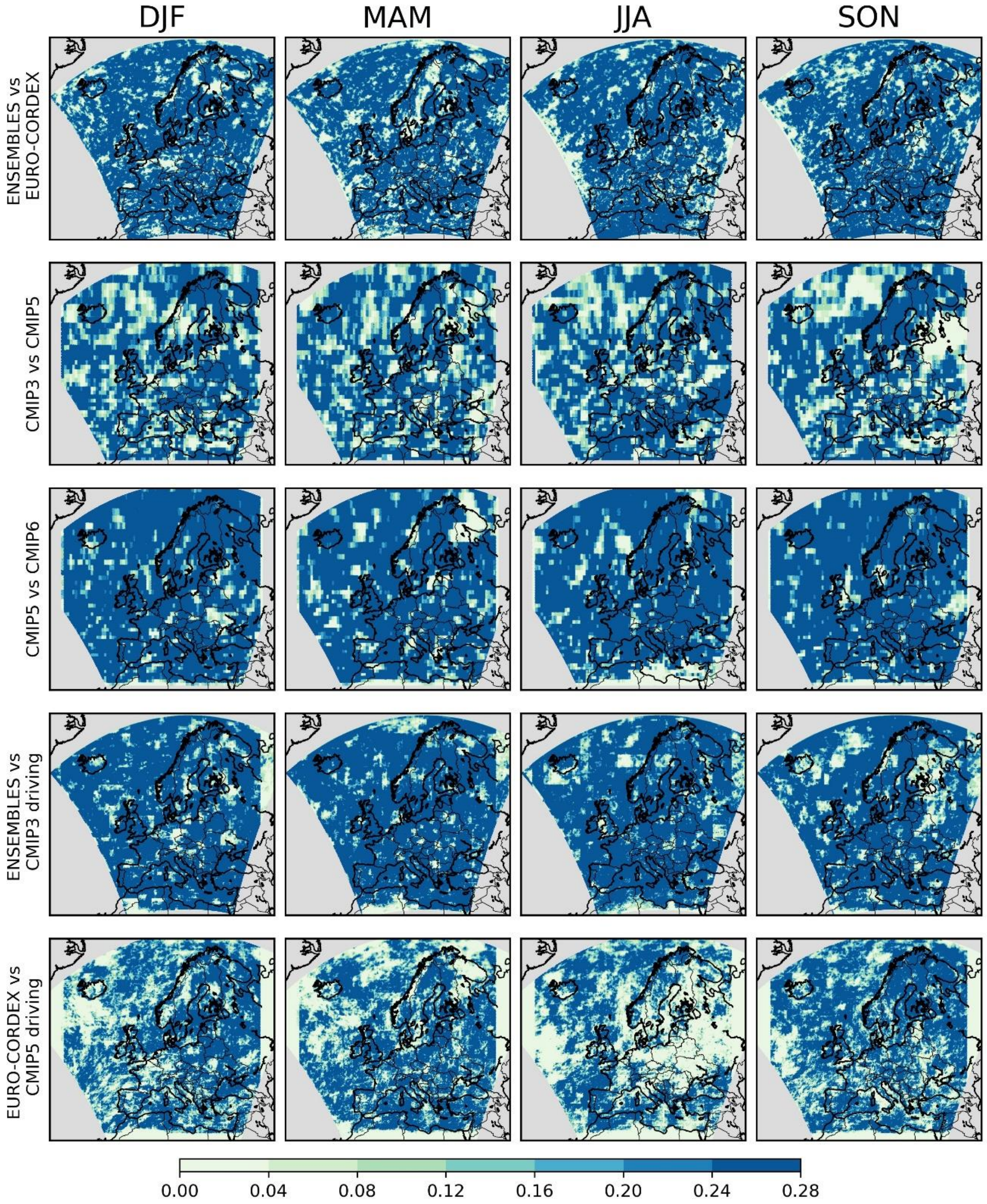

Figure S3. Same as figure S2, but for extreme precipitation.

Table S1. Chosen regional climate models of the ENSEMBLES dataset.

Institute	RCM	GCM
C4I	RCA3	HadCM3Q16
DMI	HIRHAM5	ARPEGE
DMI	HIRHAM5	BCM
DMI	HIRHAM5	ECHAM5
ETHZ	CLM	HadCM3Q0
KNMI	RACMO2	ECHAM5
KNMI	RACMO2	MIROC3.2-hires
UK MetOffice	HadRM3Q0	HadCM3Q0
UK MetOffice	HadRM3Q3	HadCM3Q3
UK MetOffice	HadRM3Q16	HadCM3Q16
MPI-M	REMO	ECHAM5
SMHI	RCA	BCM
SMHI	RCA	ECHAM5
SMHI	RCA	HadCM3Q3

Table S2. Chosen regional climate models of the EURO-CORDEX dataset.

RCM	GCM
CLMcom-CCLM4-8-17	CNRM-CERFACS-CNRM-CM5
	MOHC-HadGEM2-ES
	MPI-M-MPI-ESM-LR
CLMcom-ETH-COSMO-crCLIM-v1-1	CNRM-CERFACS-CNRM-CM5
	ICHEC-EC-EARTH
	MOHC-HadGEM2-ES
	MPI-M-MPI-ESM-LR
CNRM-ALADIN63	MOHC-HadGEM2-ES
	MPI-M-MPI-ESM-LR
	NCC-NorESM1-M
SMHI-RCA4	CNRM-CERFACS-CNRM-CM5
	ICHEC-EC-EARTH
	IPSL-IPSL-CM5A-MR
	MOHC-HadGEM2-ES
	MPI-M-MPI-ESM-LR
	NCC-NorESM1-M
DMI-HIRHAM5	CNRM-CERFACS-CNRM-CM5
	ICHEC-EC-EARTH
	IPSL-IPSL-CM5A-MR

	MOHC-HadGEM2-ES
	MPI-M-MPI-ESM-LR
	NCC-NorESM1-M
KNMI-RACMO22E	CNRM-CERFACS-CNRM-CM5
	ICHEC-EC-EARTH
	IPSL-IPSL-CM5A-MR
	MOHC-HadGEM2-ES
	MPI-M-MPI-ESM-LR
	NCC-NorESM1-M
ICTP-RegCM4-6	CNRM-CERFACS-CNRM-CM5
	MOHC-HadGEM2-ES
	MPI-M-MPI-ESM-LR
	NCC-NorESM1-M
IPSL-INERIS-WRF331F	CNRM-CERFACS-CNRM-CM5
	IPSL-CM5A-MR
	MOHC-HadGEM2-ES
	NCC-NorESM1-M
MPI-CSC-REMO2009	MPI-M-MPI-ESM-LR
RMIB-UGent-ALARO-0	CNRM-CERFACS-CNRM-CM5

Table S3. Chosen global climate models of the CMIP3 dataset.

Institute	GCM
BCCR	BCM2.0
CCCma	cgcm3-1
	cgcm3-1-t63
CNRM-CERFACS	CNRM-CM3
CSIRO-QCCCE	CSIRO-Mk3-0
	CSIRO-Mk3-5
GFDL	CM2-0
INGV	ECHAM4
IPSL	CM4
LASG-IAP	FGOALS1-0-g
MIROC	MIROC3-2-hires
	MIROC3-2-medres
MIUB	ECHO-g
MRI	Cgcm2-3-2a
NASA-GISS	AOM
NCAR	PCM1

Table S4. Chosen global climate models of the CMIP5 dataset.

GCM
ACCESS1-0
ACCESS1-3
BNU-ESM
CCSM4
CESM1-BGC
CESM1-CAM5
CNRM-CM5
CSIRO-Mk3-6-0
CanESM2
EC-EARTH
HadGEM2-AO
HadGEM2-CC
HadGEM2-ES
IPSL-CM5A-MR
IPSL-CM5B-LR
MIROC-ESM-CHEM
MIROC-ESM
MIROC5
MPI-ESM-LR
MPI-ESM-MR
MRI-CGCM3
MRI-ESM1
NorESM1-M
Inmcm4

Table S5. Chosen global climate models of the CMIP6 dataset.

Institute	GCM
AS-RCEC	TaiESM1
BCC	BCC-CSM2-MR
CAS	FGOALS-g3
CCCma	CanESM5
CCCR-IITM	IITM-ESM
CMCC	CMCC-CM2-SR5
	CMCC-ESM2
CSIRO-ARCCSS	ACCESS-CM2
CSIRO	ACCESS-ESM1-5
EC-Earth-Consortium	EC-Earth3-Veg
	EC-Earth3-Veg-LR
	EC-Earth3
	EC-Earth3-CC
INM	INM-CM5-0
	INM-CM4-8
IPSL	IPSL-CM6A-LR
KIOST	KIOST-ESM
MIROC	MIROC6
MPI-M	MPI-ESM1-2-LR
MRI	MRI-ESM2-0
NCAR	CESM2-WACCM
NCC	NorESM2-LM
	NorESM2-MM
NIMS-KMA	KACE-1-0-G
NOAA-GFDL	GFDL-CM4
	GFDL-ESM4
NUIST	NESM3

Table S6. Chosen global climate models of the HighResMIP dataset.

Institute	GCM
CNRM-CERFACS	CNRM-CM6-1-HR
EC-Earth-Consortium	EC-Earth3P-HR
MOHC	HadGEM3-GC31-HM
NERC	HadGEM3-GC31-HM
